# Evaluation of Cytoreductive Surgery With or Without Hyperthermic Intraperitoneal Chemotherapy for Stage III Epithelial Ovarian Cancer

**DOI:** 10.1001/jamanetworkopen.2020.13940

**Published:** 2020-08-25

**Authors:** Ziying Lei, Yue Wang, Jiahong Wang, Ke Wang, Jun Tian, Ying Zhao, Lipai Chen, Jin Wang, Jiali Luo, Manman Jia, Hongsheng Tang, Qingjun He, Quanxing Liao, Xiansheng Yang, Tianpei Guan, Li Wang, Shuzhong Cui

**Affiliations:** 1 Affiliated Cancer Hospital and Institute of Guangzhou Medical University, Guangzhou, China; 2 Henan Provincial People’s Hospital, Zhengzhou, China; 3 Tianjin Medical University Cancer Institute and Hospital, National Clinical Research Center of Cancer, Tianjin, China; 4 Huaihe Hospital of Henan University, Kaifeng, China; 5 Affiliated Tumor Hospital of Zhengzhou University, Tumor Hospital of Henan Province, Henan, China

## Abstract

**Importance:**

Interval cytoreductive surgery combined with hyperthermic intraperitoneal chemotherapy (HIPEC) showed promising oncologic outcomes for patients with advanced ovarian cancer, but a large-scale, multicenter study to evaluate the efficacy of HIPEC combined with primary cytoreductive surgery (PCS) has yet to be conducted.

**Objective:**

To compare survival outcomes between PCS with HIPEC vs PCS alone for patients with stage III epithelial ovarian cancer.

**Design, Setting, and Participants:**

This cohort study was conducted from January 2010 to May 2017 at 5 high-volume institutions in China. A total of 584 patients with stage III primary epithelial ovarian cancer were treated with either PCS alone or PCS with HIPEC. The median (interquartile range) follow-up period was 42.2 (33.3-51.0) months. Data analysis was conducted from August to December 2019.

**Exposures:**

PCS with HIPEC vs PCS alone.

**Main Outcomes and Measures:**

Primary outcomes were median survival time and 3-year overall survival. The inverse probability of treatment weighting (IPTW) method, based on propensity score, was used to control for confounding factors.

**Results:**

From a total of 789 patients with stage III epithelial ovarian cancer, 584 patients (74.0%; mean [SD] age, 55.0 [10.5] years) were ultimately included for IPTW in this study. Of the 584 patients, 425 (72.8%) underwent PCS with HIPEC and 159 (27.2%) underwent PCS alone. After IPTW adjustment, the median survival time was 49.8 (95% CI, 45.2-60.2) months for patients undergoing PCS with HIPEC and 34.0 (95% CI, 28.9-41.5) months for patients undergoing PCS alone, and the 3-year overall survival rate was 60.3% (95% CI, 55.3%-65.0%) for patients undergoing PCS with HIPEC and 49.5% (95% CI, 41.0%-57.4%) for patients undergoing PCS alone (weighted hazard ratio, 0.64; 95% CI, 0.50-0.82; *P* < .001). Further stratified into complete and incomplete surgery subgroups, patients in the PCS with HIPEC group had significantly better survival than those in the PCS group, except for the 3-year overall survival rate in the incomplete subgroup. Among those who underwent complete surgical procedures and comparing those who received PCS with HIPEC vs those who received PCS alone, the median survival time was 53.9 (95% CI, 46.6-63.7) months vs 42.3 (95% CI, 31.1-59.3) months (*P* = .02), and the 3-year overall survival rate was 65.9% (95% CI, 60.1%-71.2%) vs 55.4% (95% CI, 44.7%-64.8%) (*P* = .04); meanwhile, among patients who underwent incomplete surgical procedures and comparing those who received PCS with HIPEC vs those who received PCS alone, the median survival time was 29.2 (95% CI, 22.3-45.5) months vs 19.9 (95% CI, 11.6-39.1) months (*P* = .03), and the 3-year OS rate was 44.3% (95% CI, 34.6%-53.4%) vs 36.7% (95% CI, 23.4%-50.1%) (*P* = .19). The treatment was well tolerated in both groups.

**Conclusions and Relevance:**

In this study, the PCS with HIPEC treatment approach was associated with better long-term survival. When complete PCS is possible, this approach could be a valuable therapy for patients with stage III epithelial ovarian cancer.

## Key Points

**Question:**

Is the addition of hyperthermic intraperitoneal chemotherapy (HIPEC) to primary cytoreductive surgery (PCS) associated with better outcomes for patients with stage III epithelial ovarian cancer?

**Findings:**

In this multicenter retrospective cohort study of 584 patients with stage III epithelial ovarian cancer, for patients undergoing PCS with HIPEC and those undergoing PCS alone, the median overall survival was 49.8 and 34.0 months, respectively, and the 3-year overall survival rates were 60.3% and 49.5%, respectively. Complete PCS with HIPEC was associated with the best survival outcomes, with a median overall survival of 53.9 months and a 3-year overall survival rate of 65.9%.

**Meaning:**

In this study, the addition of HIPEC to PCS was associated with better survival outcomes for patients with stage III epithelial ovarian cancer.

## Teaser

This cohort study compares survival outcomes between primary cytoreductive surgery with hyperthermic intraperitoneal chemotherapy (HIPEC) vs primary cytoreductive surgery alone for patients with stage III epithelial ovarian cancer.

## Introduction

Ovarian cancer is the leading cause of death among all gynecological malignant neoplasms. In nearly 70% of cases, lesions have already spread beyond the ovaries to the peritoneal cavity by the time of initial diagnosis.^1,2^ Currently, optimal primary cytoreductive surgery (PCS) with platinum/paclitaxel-based intravenous chemotherapy is the standard treatment for surgical candidates.^3,4^ However, some randomized clinical trials (RCTs) have recommended neoadjuvant chemotherapy and interval debulking surgery as the standard clinical choice.^4,5,6^ Additionally, intraperitoneal chemotherapy has significant advantages over intravenous chemotherapy. However, most patients developed catheter-related complications. These discrepancies have initiated a debate and hampered the widespread development of intraperitoneal chemotherapy.^7^ Therefore, novel and more tolerable therapeutic alternatives to intraperitoneal chemotherapy that could prolong survival outcomes also need to be considered.

Theoretically, the permeability of anticancer drugs is improved by intraperitoneal delivery combined with hyperthermia, which increases the accumulation of the drug in the cancer cells. Moreover, as a consequence of the loss of cellular DNA repair capacity, the cytotoxic effect appears to be amplified, leading to higher sensitivity to chemotherapy.^8,9^ Many studies have suggested that cytoreductive surgery followed by hyperthermic intraperitoneal chemotherapy (HIPEC) could prolong the overall survival (OS) of patients with advanced ovarian cancer.^10,11,12,13^

Van Driel et al^6^ presented the first RCT of HIPEC for stage III epithelial ovarian cancer, finding HIPEC to have a noticeable survival benefit. In this trial, all patients underwent 3 cycles of preoperative intravenous chemotherapy, which resulted in a satisfactory debulking surgery rate of 98%. The report by van Driel et al^6^ suggested that neoadjuvant chemotherapy could serve a critical role in the treatment of bulky advanced ovarian cancer. However, a meta-analysis of 22 cohorts involving 835 patients with advanced ovarian cancer^14^ demonstrated that neoadjuvant chemotherapy is associated with worse prognosis. There is presently a lack of large-scale, multicenter RCTs comparing the survival outcomes between PCS with and without HIPEC for patients with advanced epithelial ovarian cancer.

Despite the existence of high-quality evidence supporting the use of HIPEC in the treatment of patients with advanced ovarian cancer, its widespread adoption, and the National Comprehensive Cancer Network 2019 proposing it as an interval debulking surgery,^15^ to our knowledge, no current multicenter, large-scale studies have examined whether a combination of PCS with HIPEC can improve the prognosis of patients with epithelial ovarian cancer. Thus, in the current study, we used data from 584 patients in 5 high-volume institutions to evaluate the efficacy and safety of PCS with HIPEC and PCS without HIPEC for patients with stage III epithelial ovarian cancer.

## Methods

### Patient Population

**Figure 1.**
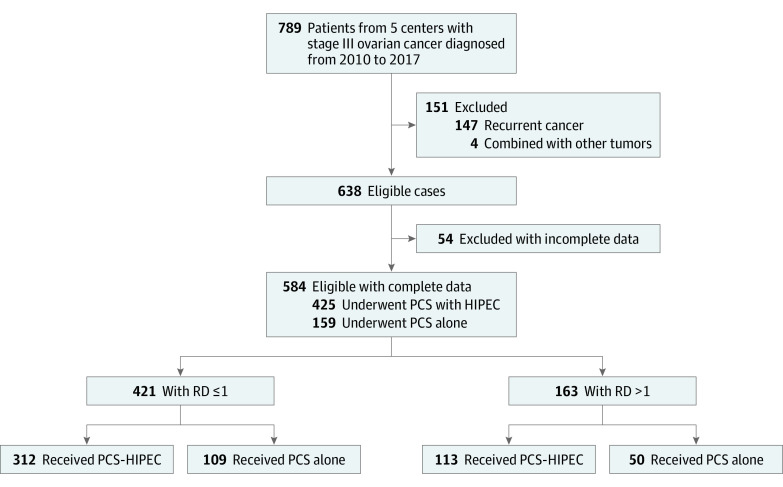
Flowchart Showing the Selection Process for Patients With Stage III Ovarian Cancer PCS indicates primary cytoreductive surgery; HIPEC, hyperthermic intraperitoneal chemotherapy; and RD, residual disease.

This multicenter retrospective study was conducted by 5 members of the Chinese Peritoneal Oncology Study group. The purpose of this group is to create a multicenter HIPEC database for the treatment of ovarian, colorectal, pancreatic, and gastric cancer. Between January 2010 and May 2017, a total of 789 patients with stage III epithelial ovarian cancer were treated in the 5 participating centers (Figure 1). After the exclusion of 147 patients with recurrent disease, 4 patients with epithelial ovarian cancer combined with other tumor types, and 54 patients with incomplete data, 584 patients (74.0%) with stage III primary epithelial ovarian cancer were enrolled. The inclusion criteria were as follows: (1) histologically confirmed diagnosis of epithelial ovarian cancer, (2) no antitumor treatment before the operation, and (3) no evidence of extra-abdominal metastasis. This study was approved by the institutional review board and the ethics committee of each participating center. Written informed consent was waived because of the retrospective design. Our study followed the Strengthening the Reporting of Observational Studies in Epidemiology (STROBE) reporting guideline.

### Surgical Procedures and HIPEC

The completeness of the cancer resection was assessed according to the residual disease classification. Complete surgery was defined as residual disease measuring 1.0 cm or less in diameter and incomplete surgery as the presence of 1 or more residual nodules measuring greater than 1.0 cm in diameter.

HIPEC was simultaneously administered at the end of PCS using the closed technique. Two inflow catheters were positioned in the pelvic cavity, and 2 outflow catheters were positioned in the hepatorenal recess and hepatosplenic recess, respectively. Constant, precise temperature control was obtained with a continuous closed loop with a circulating pump, water-bath heater, heat exchanger, and real-time temperature monitoring. HIPEC was performed at a flow rate of 400 to 600 mL/min and a perfusion volume of 2 L/m^2^ saline for 60 minutes using the BR-TRG-I hyperthermic perfusion treatment technique (Bright Medical Tech). Circulating heated saline with cisplatin at a dose of 50 mg/m^2^ was kept within 0.1 °C of 43 °C. The HIPEC procedure was recommended to be performed on day 1, day 3, and day 5. The mean (SD) number of HIPEC treatments was 2.8 (0.8).

### Follow-up

The primary outcomes were median survival time (MST) and 3-year overall survival (OS). The cutoff date for follow-up was June 2019. We defined OS as the time between PCS until death from any cause. Most complete survival data were collected from the outpatient follow-up. The patients were followed up through telephone calls and messages from the department responsible for follow-up at each of the participating institutions. Information from patients who were alive at the cutoff date or who were uncontactable was defined as censored data. Adverse events were graded according to the National Cancer Institute Common Terminology Criteria for Adverse Events version 4.0.

### Statistical Analysis

This study was designed to evaluate the efficacy and safety of PCS with HIPEC vs PCS alone on the OS of patients with stage III epithelial ovarian cancer. Continuous variables were described as mean with SD or median with interquartile range (IQR) and were compared using the *t* test or Mann-Whitney U test. Categorical variables were expressed as frequency and percentage and were compared using Pearson χ^2^ test or Fisher exact test.

The inverse probability of treatment weighting (IPTW) model was performed to control for differences in baseline characteristics between the PCS with HIPEC and PCS groups. The propensity score was evaluated as the probability of receiving HIPEC for each patient using a multivariate logistic regression model that included 2 major confounding factors: ascites and cycles of chemotherapy. These 2 variables were selected via a stepwise method that included the following factors: age, Eastern Cooperative Oncology Group performance status, grade, histology, ascetic fluid, and cycles of chemotherapy. Based on the propensity score, a stabilized inverse-probability weight was calculated for each patient as follows: for HIPEC patients, the proportion of treatment was divided by the propensity score; for non-HIPEC patients, 1 − the proportion of treatment was divided by 1 − the propensity score.

The primary outcome was survival. Survival curves, survival rates, and MST were generated using the Kaplan-Meier method, and differences between curves were estimated with the log rank test. A Cox proportional hazards model was used to calculate hazard ratios (HRs) as indicators of mortality risk. Subgroup analysis was performed to assess the consistency of the association of HIPEC with outcomes across subgroups, and interactions were evaluated. The analytical approaches used in the subgroup analysis were consistent with the overall analysis. These analyses were performed before and after weighting. In the weighted analysis, the weighted Kaplan-Meier method, log rank test, and Cox proportional hazards model were used to create adjusted results.

The statistical software package R version 3.6.1 (R Project for Statistical Computing) and SPSS statistics software version 24.0 (IBM Corp) were used for the statistical analysis. A 2-sided *P* < .05 was considered statistically significant.

## Results

### Patients

A total of 584 patients, with a mean (SD) age of 55.0 (10.5) years were included in the study. Of these patients, 425 (72.8%) underwent PCS with HIPEC and 159 (27.2%) underwent PCS alone (Figure 1). Complete surgery (residual lesions measuring ≤1.0 cm in diameter) was received by 421 patients (72.1%), and the other 163 patients (27.9%) underwent incomplete surgery.

The baseline demographic and clinical characteristics of the patients before and after IPTW adjustment are shown in Table 1. Before IPTW, compared with the PCS group, patients in the PCS with HIPEC group had more frequently completed at least 6 cycles of chemotherapy (56 [35.2%] vs 220 [51.8%]; *P* = .03) and had a more moderate and large ascites (moderate: 19 [11.9%] vs 75 [17.6%]; large: 38 [23.9%] vs 125 [29.4%]; *P* = .06). IPTW based on propensity score was performed to control for potential confounding effects. Except for hospital stay and time to first flatus, there were no statistical differences in baseline characteristics between the groups after IPTW adjustment.

**Table 1.** Clinical and Clinicopathological Parameters of Participants

Characteristic	Patients, No. (%)			*P* value	PS-weighted *P* value
	All (N = 584)	Receiving PCS with HIPEC (n = 425)	Receiving PCS (n = 159)		
Age, mean (SD), y	55.0 (10.5)	55.1 (10.1)	54.6 (11.7)	.68	.32
ECOG PS					
0 or 1	575 (98.5)	419 (98.6)	156 (98.1)	.97	.62
2 or 3	9 (1.5)	6 (1.4)	3 (1.9)		
Histology					
Serous	536 (91.8)	390 (91.8)	146 (91.8)	.47	.32
Mucinous	24 (4.1)	18 (4.2)	6 (3.8)		
Endometrioid	6 (1.0)	3 (0.7)	3 (1.9)		
Clear-cell	10 (1.7)	9 (2.1)	1 (0.6)		
Other	8 (1.4)	5 (1.2)	3 (1.9)		
Grade					
High	534 (91.4)	385 (90.6)	149 (93.7)	.23	.17
Low	50 (8.6)	40 (9.4)	10 (6.3)		
Ascetic fluid amount^a^					
None	179 (30.7)	119 (28.0)	60 (37.7)	.06	.41
Little	148 (25.3)	106 (24.9)	42 (26.4)		
Moderate	94 (16.1)	75 (17.6)	19 (11.9)		
Large	163 (27.9)	125 (29.4)	38 (23.9)		
Residual disease					
≤1 cm	421 (72.1)	312 (73.4)	109 (68.6)	.24	.20
>1 cm	163 (27.9)	113 (26.6)	50 (31.4)		
Cycles of chemotherapy					
0	87 (14.9)	59 (13.9)	28 (17.6)	.03	.90
1	81 (13.9)	52 (12.2)	29 (18.2)		
2	45 (7.7)	28 (6.6)	17 (10.7)		
3	29 (5.0)	19 (4.5)	10 (6.3)		
4	36 (6.2)	26 (6.1)	10 (6.3)		
5	30 (5.1)	21 (4.9)	9 (5.7)		
≥6	276 (47.3)	220 (51.8)	56 (35.2)		
Clinical characteristics, mean (SD)					
Time to first flatus, d	4.2 (2.1)	4.3 (2.2)	4.0 (1.8)	.04	.04
Hospital stay, d	12.8 (5.1)	13.8 (5.1)	10.0 (3.8)	<.001	<.001

Abbreviations: ECOG PS, Eastern Cooperative Oncology Group performance status; HIPEC, hyperthermic intraperitoneal chemotherapy; PCS, primary cytoreductive surgery; PS, propensity score.

^a^ Ascitic fluid categories as follows: little, below the pelvis; moderate, above the pelvic cavity; large, throughout the abdominal cavity.

### Safety

The postoperative adverse events are listed in Table 2. The most common grade 3 or 4 adverse events were electrolyte disturbance, anemia, leukopenia, and neutropenia in both groups. Grade 3 or 4 electrolyte disturbance occurred more frequently in the PCS with HIPEC group than the PCS alone group (118 [28.1%] vs 18 [11.5%]; *P* < .001). No significant differences were observed in nonhematologic toxic effects between the 2 groups. The PCS group had shorter mean (SD) hospital stays and time to first flatus than the PCS with HIPEC group (10.0 [3.8] days vs 13.8 [5.1] days; *P* < .001; 4.0 [1.8] days vs 4.3 [2.2] days; *P* = .04) (Table 1). No other severe treatment-related complications occurred.

**Table 2.** Major Toxic Effects and Complications in the Ovarian Cancer Patients^a^

Adverse event	Patients, No. (%)								*P* value	PS-weighted *P* value
	Receiving PCS with HIPEC (n = 425)				Receiving PCS alone (n = 159)					
	Untested	Grade 1-2	Grade 3	Grade 4	Untested	Grade 1-2	Grade 3	Grade 4		
Hypoalbunemia	17 (4.0)	381 (93.4)	15 (3.7)	0	14 (8.8)	123 (84.8)	3 (2.1)	0	<.001	.001
Anemia	4 (0.9)	309 (73.4)	87 (20.7)	0	4 (2.5)	117 (75.5)	21 (13.5)	0	.03	.10
Nausea	1 (0.2)	298 (70.3)	0	0	2 (1.3)	79 (50.3)	0	0	<.001	<.001
Vomiting	1 (0.2)	257 (60.6)	0	0	2 (1.3)	64 (40.8)	0	0	<.001	<.001
Electrolyte disturbance^b^	5 (1.2)	250 (59.5)	109 (26.0)	9 (2.1)	3 (1.9)	70 (44.9)	14 (9.0)	4 (2.6)	<.001	<.001
Abdominal bloating	1 (0.2)	201 (47.4)	0	0	2 (1.3)	79 (50.3)	0	0	.53	.39
Fever	1 (0.2)	175 (41.3)	3 (0.7)	0	2 (1.3)	55 (35.0)	2 (1.3)	0	.34	.33
Leukopenia	4 (0.9)	148 (35.2)	70 (16.6)	30 (7.1)	4 (2.5)	40 (25.8)	19 (12.3)	9 (5.8)	.02	.22
Abdominal pain	1 (0.2)	148 (34.9)	4 (0.9)	0	2 (1.3)	69 (43.9)	3 (1.9)	0	.08	.03
Neutropenia	4 (0.9)	119 (28.3)	65 (15.4)	61 (14.5)	4 (2.5)	40 (25.8)	14 (9.0)	16 (10.3)	.03	.37
AST increased	11 (2.6)	88 (21.3)	3 (0.7)	0	6 (3.8)	28 (18.3)	1 (0.7)	0	.73	.96
ALT increased	11 (2.6)	77 (18.6)	4 (1.0)	0	6 (3.8)	24 (15.7)	0	0	.20	.46
Thrombocytopenia	4 (0.9)	73 (17.3)	14 (3.3)	2 (0.5)	4 (2.5)	22 (14.2)	6 (3.9)	0	.75	.77
Pulmonary infection	1 (0.2)	52 (12.3)	0	0	1 (0.6)	16 (10.1)	1 (0.6)	0	.26	.23
Diarrhea	1 (0.2)	36 (8.5)	1 (0.2)	0	2 (1.3)	10 (6.4)	2 (1.3)	0	.20	.44
Pleural effusion	1 (0.2)	23 (5.4)	0	0	1 (0.6)	6 (3.8)	0	0	.42	.67
Creatinine increased	11 (2.6)	22 (5.3)	2 (0.5)	0	5 (3.1)	2 (1.3)	0	0	.07	.03
Constipation	1 (0.2)	17 (4.0)	0	0	2 (1.3)	10 (6.4)	0	0	.23	.20
Neurotoxicity	1 (0.2)	11 (2.6)	0	0	2 (1.3)	2 (1.3)	0	0	.52	.59
Ileus	1 (0.2)	7 (1.7)	0	0	2 (1.3)	6 (3.8)	0	0	.21	.21
Thromboembolic event	1 (0.2)	2 (0.5)	0	0	2 (1.3)	0	0	0	.99	.39
Incisional infection	1 (0.2)	1 (0.2)	0	0	4 (2.5)	0	0	0	.99	.54
Adhesions	1 (0.2)	1 (0.2)	0	0	2 (1.3)	1 (0.6)	0	0	.47	.21
Dyspnea	1 (0.2)	1 (0.2)	1 (0.2)	0	1 (0.6)	2 (1.3)	1 (0.6)	0	.16	.16
Cardiotoxicity	1 (0.2)	1 (0.2)	0	0	2 (1.3)	1 (0.6)	0	0	.47	.47
Rash	1 (0.2)	1 (0.2)	0	0	2 (1.3)	2 (1.3)	0	0	.18	.09
Anastomotic leakage	1 (0.2)	0	0	0	4 (2.5)	1 (0.6)	0	0	.27	.10
Pancreatitis	1 (0.2)	0	1 (0.2)	0	4 (2.5)	0	0	0	.99	.54
Intra-abdominal infection	1 (0.2)	0	0	0	4 (2.5)	1 (0.6)	0	0	.27	.10
Intra-abdominal bleeding	1 (0.2)	0	1 (0.2)	0	4 (2.5)	1 (0.6)	0	0	.46	.16
ARDS	1 (0.2)	0	0	1 (0.2)	1 (0.6)	0	0	0	.99	.54

Abbreviations: ALT, alanine aminotransferase; ARDS, acute respiratory distress syndrome; AST, aspartate aminotransferase; HIPEC, hyperthermic intraperitoneal chemotherapy; PCS, primary cytoreductive surgery; PS, propensity score.

^a^ According to Common Terminology Criteria for Adverse Events version 4.

^b^ Electrolyte disturbances including hypokalemia, hyperkalemia, hyponatremia, hypernatremia, hypomagnesemia, hypophosphatemia, and hypercalcemia.

### Efficacy

**Figure 2.**
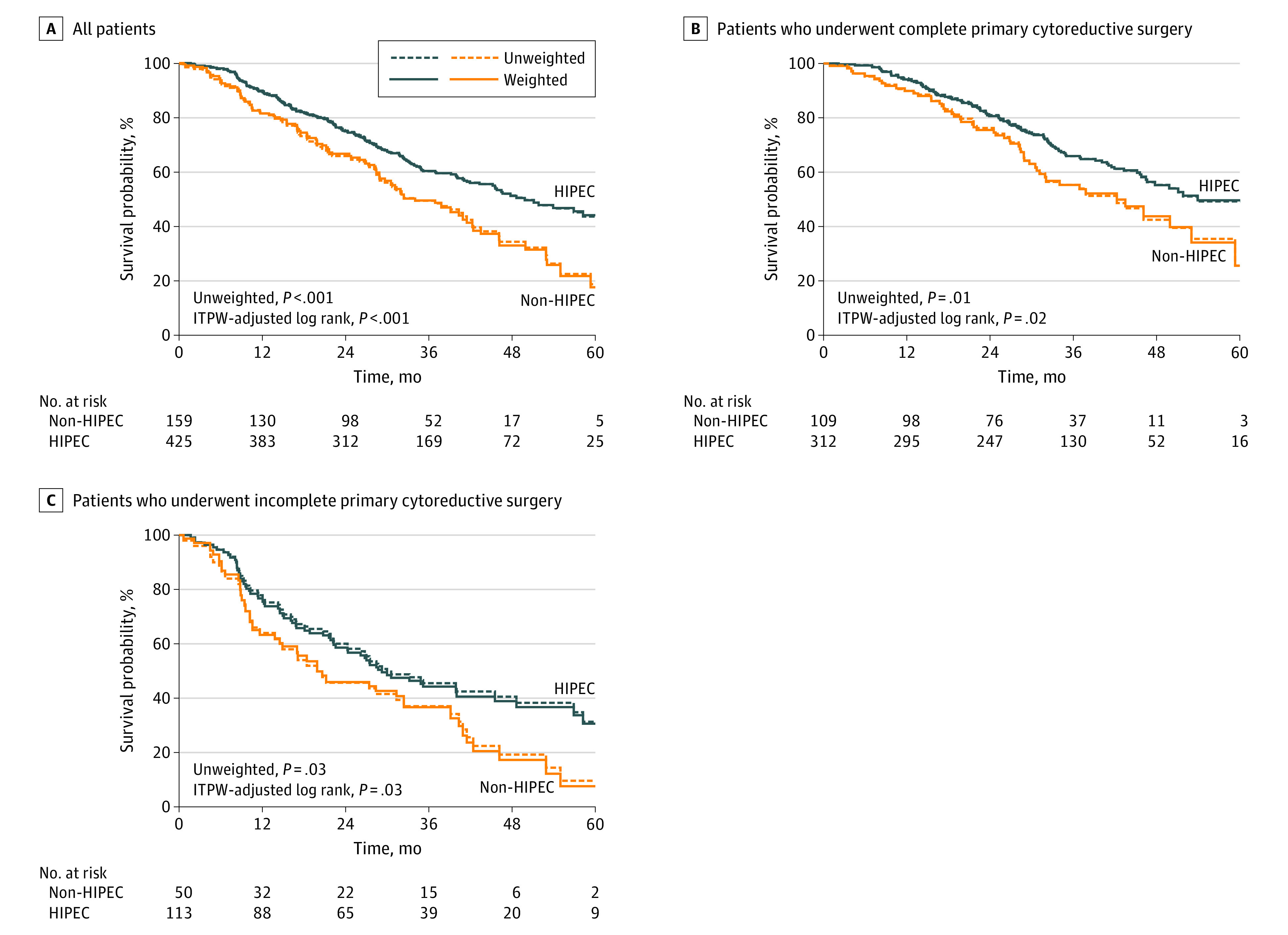
Kaplan-Meier Estimates of Overall Survival Before and after the inverse probability of treatment weighting (IPTW) analysis for all patients with stage III ovarian cancer and divided into groups of patients who underwent complete primary cytoreductive surgery and who underwent incomplete primary cytoreductive surgery. HIPEC indicates hyperthermic intraperitoneal chemotherapy.

The median (interquartile range) follow-up period was 42.2 (33.3-51.0) months. Survival analysis revealed that patients with epithelial ovarian cancer in the PCS with HIPEC group had a significantly higher median OS than those in the PCS alone group. The MST was 49.8 (95% CI, 45.2-60.2) months in the PCS with HIPEC group vs 34.0 (95% CI, 28.9-42.3) months in the PCS group (HR, 0.63; 95% CI, 0.49-0.82; *P* < .001). The 3-year OS rate was 60.5% (95% CI, 55.5%-65.2%) for the PCS with HIPEC group vs 49.6% (95% CI, 41.2%-57.5%) for the PCS alone group. After IPTW adjustment, PCS with HIPEC was still associated with better oncologic outcomes, with an MST of 49.8 (95% CI, 45.2-60.2) months in the PCS with HIPEC group and 34.0 (95% CI, 28.9-41.5) months in the PCS alone group (weighted HR, 0.64; 95% CI, 0.50-0.82; *P* < .001) (Figure 2A). The 3-year OS rate was 60.3% (95% CI, 55.3%-65.0%) in the PCS with HIPEC group and 49.5% (95% CI, 41.0%-57.4%) in the PCS alone group (*P* = .01).

**Figure 3.**
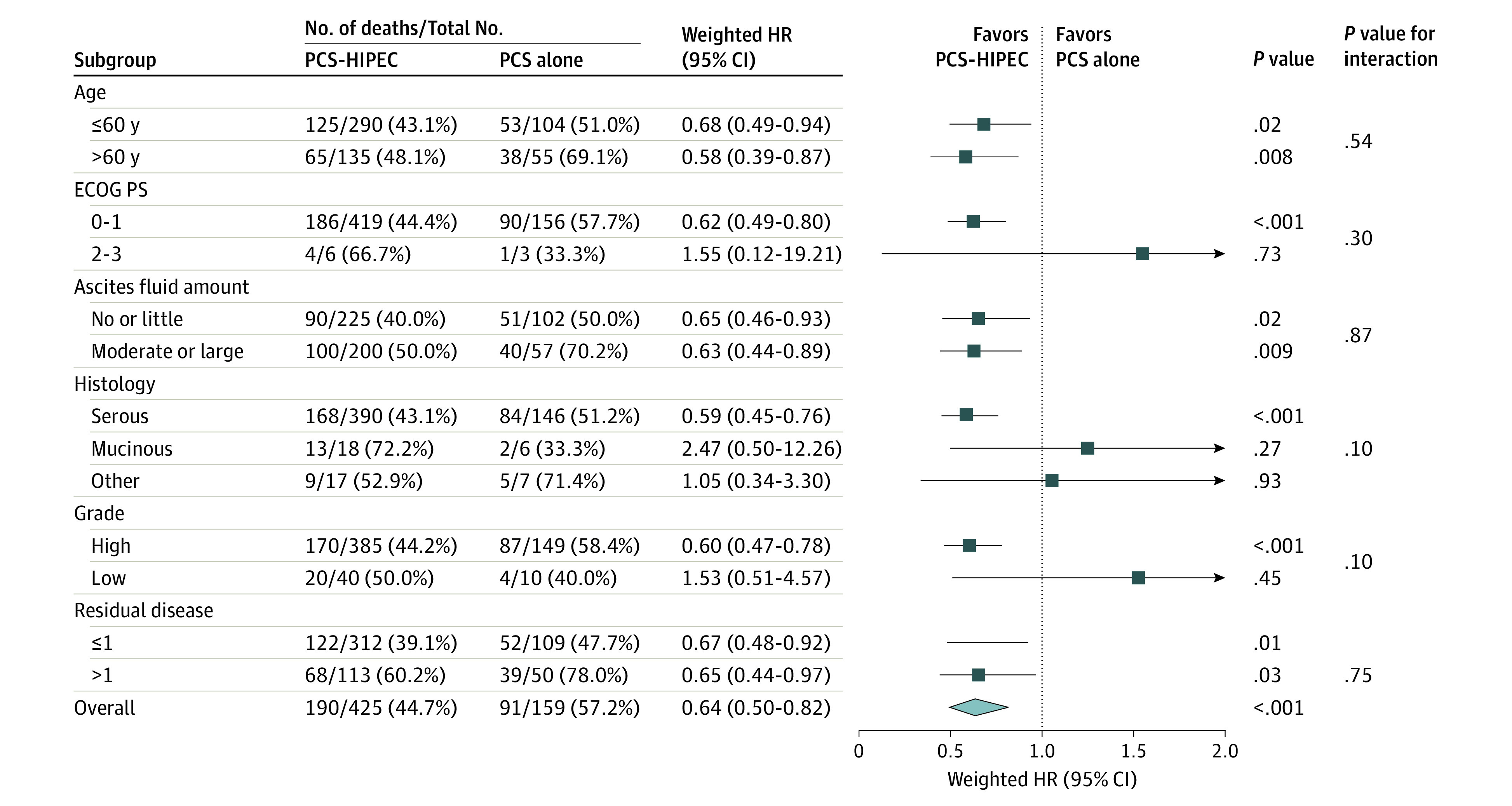
Subgroup Analyses of Overall Survival A forest plot of showing the association of hyperthermic intraperitoneal chemotherapy (HIPEC) with overall survival in patient subgroups. HR indicates hazard ratio; PCS, primary cytoreductive surgery; ECOG PS, Eastern Cooperative Oncology Group performance status.

After further stratification into subgroups who received complete and incomplete surgery, the addition of HIPEC also had significantly better survival outcomes than surgery alone. In the complete surgery subgroup, the median OS was 53.9 (95% CI, 46.6-63.7) months for the PCS with HIPEC group and 42.3 (95% CI, 31.1-59.3) months for the PCS alone group (weighted HR, 0.67; 95% CI, 0.48-0.92; *P* = .02) (Figure 2B). The 3-year OS rate was 65.9% (95% CI, 60.1%-71.2%) in the PCS with HIPEC group and 55.4% (95% CI, 44.7%-64.8%) in the PCS alone group (*P* = .04). In the incomplete surgery subgroup, the median OS was 29.2 (95% CI, 22.3-45.5) months for the PCS with HIPEC group and 19.9 (95% CI, 11.6-39.1) months with PCS alone (weighted HR, 0.65; 95% CI, 0.44-0.97; *P* = .03) (Figure 2C). The 3-year OS rate was 44.3% (95% CI, 34.6%-53.4%) in the PCS with HIPEC group and 36.7% (95% CI, 23.4%-50.1%) in the PCS alone group, but the difference was not statistically significant (*P* = .19). The association of HIPEC was consistent across the levels of stratification factors. For example, patients aged 60 years or younger who received PCS with HIPEC compared with those who received PCS alone had an HR of 0.68 (95% CI, 0.49-0.94; *P* = .02), while those older than 60 years in the PSC with HIPEC group vs the PCS alone group had an HR of 0.58 (95% CI, 0.39-0.87; *P* = .008) (Figure 3). No significant interaction was found (Figure 3).

## Discussion

In this study, we evaluated adjuvant HIPEC with PCS for the treatment of advanced epithelial ovarian cancer. Our findings showed that for women with stage III epithelial ovarian cancer, the survival outcomes were significantly better for patients receiving PCS with HIPEC than for those receiving PCS without HIPEC (median OS, all patients: 49.8 [95% CI, 45.2-60.2] months vs 34.0 [95% CI, 28.9-41.5] months; patients who received complete surgery: 53.9 [95% CI, 46.6-63.7] months vs 42.3 [95% CI, 31.1-59.3] months; patients who received incomplete surgery: 29.2 [95% CI, 22.3-45.5] months vs 19.9 [95% CI, 11.6-39.1] months). Furthermore, when complete surgery was possible, the PCS with HIPEC approach achieved the best survival outcomes.

Nearly 70% of patients with advanced ovarian cancer relapse and die of the disease, even after standard-of-care treatment according to clinical guidelines.^16^ Given that the peritoneal cavity is the primary site of tumor invasion and can be continuously exposed to a high concentration of chemotherapeutic agents, it seems rational to administer intraperitoneal chemotherapy directly into the peritoneum.^7,17,18^ An earlier Gynecologic Oncology Group–172 trial demonstrated the benefits of adjuvant intraperitoneal chemotherapy and showed that the median OS was 16 months longer in the experimental group compared with the standard group among patients with optimally debulked stage III ovarian cancer (median OS, 65.6 months vs 49.7 months). However, only 42% of patients completed all of the recommended 6 cycles of intraperitoneal cisplatin and paclitaxel, mainly because poor tolerance and increased toxic effects limited further clinical practice.^7^

Treatment with PCS followed by systemic chemotherapy has been the standard of care for newly advanced ovarian cancer for many years. However, neoadjuvant chemotherapy followed by interval cytoreductive surgery has emerged as an alternative strategy, given that it has demonstrated comparable survival to PCS.^4,5^ Furthermore, the decision between PCS and neoadjuvant chemotherapy followed by interval cytoreductive surgery remains controversial, owing to unsatisfactory optimal cytoreduction rates and a poor median OS.^3^ Maximum surgical debulking, especially when complete resection of the disease is possible, is undoubtedly considered the most crucial prognostic factor for patients with advanced ovarian cancer.^19,20^ Indeed, in our study, complete PCS followed by HIPEC was associated with the best survival outcomes, with a median OS of 53.9 (95% CI, 46.6-63.7) months and a 3-year OS rate of 65.9% (95% CI, 60.1%-71.2%). However, the complete primary cytoreduction rate of 72.1% was relatively lower than that of the patients administered 3 cycles of neoadjuvant chemotherapy in the RCT,^6^ further demonstrating the importance of neoadjuvant chemotherapy for increasing the rate of complete surgery.

In recent decades, the strategy of cytoreductive surgery plus HIPEC in treating advanced ovarian cancer has attracted much attention.^21,22,23,24,25^ Research has shown that cytoreductive surgery with HIPEC can significantly improve survival in most peritoneal carcinomas from gastric cancer,^26^ colorectal carcinomatosis,^27,28^ and pseudomyxoma peritonei.^29^ Furthermore, previous studies have confirmed that cytoreductive surgery plus HIPEC is effective and could improve the prognosis for patients with advanced ovarian cancer.^10,11,13^ Recently, the addition of HIPEC to interval cytoreductive surgery has led to significantly better survival outcomes among patients with stage III epithelial ovarian cancer (median recurrence-free survival, 14.2 months vs 10.7 months; median OS, 45.7 months vs 33.9 months).^6^ Accordingly, this conclusion has been adopted in the latest version of the National Comprehensive Cancer Network guidelines for ovarian cancer, which recommend that HIPEC with cisplatin can be considered at the time of interval cytoreductive surgery for stage III disease.^15^ Despite this, further randomized clinical trials investigating whether the addition of HIPEC to PCS is also effective have yet to be conducted. Our results found that the median OS was 15.8 months longer among patients who underwent PCS combined with HIPEC than among those who underwent PCS without HIPEC. In the complete surgery and incomplete surgery subgroups, HIPEC therapy maintained its association with better OS. This success warrants further confirmation in prospective, multicenter, open-label, randomized, phase 3 trials of PCS with or without HIPEC.

The performance of HIPEC is heterogeneous, which may interfere with the results of these trials for patients with advanced ovarian cancer. Variable factors include the method of HIPEC used, treatment temperature, and frequency of treatment.^30^ Furthermore, we are unsure whether HIPEC performed at 40 °C for 1 cycle is the best regimen. In our current study, HIPEC was performed with the same chemotherapy drug (cisplatin, 50 mg/m^2^), temperature (within 0.1 °C of 43 °C), and time (60 minutes), and it was performed repeatedly (mean [SD], 2.8 [0.8] times), which might have further increased the efficacy. Intraperitoneal chemotherapy can achieve high response rates in the abdomen because the peritoneal-plasma barrier offers dose-intensive therapy.^31^ High concentrations of chemotherapeutics could directly act on tumor cells. Hyperthermia has been demonstrated to enhance the cytotoxicity of chemotherapeutic drugs and promote their infiltration in tumor cells, which suggests that hyperthermia might increase the beneficial effects of chemotherapeutic drugs.^31,32,33^ Our results showed that PCS plus HIPEC resulted in longer OS than PCS alone.

Our findings and previous observations indicate that the addition of HIPEC could improve OS for patients with advanced ovarian cancer. Additionally, a French multicenter retrospective cohort study showed that a curative therapeutic approach combining cytoreductive surgery and HIPEC could improve the long-term oncologic outcomes in patients with advanced ovarian cancer, even in patients with chemo-resistant disease.^10^ Another study revealed that the treatment of microscopic disease following cytoreductive surgery plus HIPEC in patients with advanced ovarian cancer is valid and can prolong disease-free survival.^11^ Importantly, another prospective randomized phase 3 study on cytoreductive surgery plus HIPEC involving 120 patients with advanced ovarian cancer showed that the 3-year OS rates of the HIPEC group and the non-HIPEC group were 75% and 18%, respectively, and the median OS in the HIPEC group was prolonged by 13 months.^34^

The rates of postoperative complications were similar between the PCS with HIPEC and PCS groups. The addition of HIPEC had little association with postoperative toxic events or on grade 3 and 4 adverse events, except electrolyte disturbance. HIPEC did not result in higher morbidity and mortality and was still within the range reported in the review, but the time to first flatus and length of hospital stay were longer.^35^

### Limitations

There are several limitations to the present study. First, the main limitation is the study’s retrospective design, which brings selection bias, detection bias, and analysis bias. Second, homogeneity might have been lacking in the selection of patients undergoing PCS, PCS with HIPEC, or neoadjuvant chemotherapy in the participating institutions. The study also showed inferior survival compared with the Gynecologic Oncology Group–172 trial,^7^ which might be because of the relatively higher rate of incomplete surgery in our study. Finally, this study did not demonstrate statistical superiority of adjuvant HIPEC in terms of the 3-year OS rate of patients who underwent incomplete surgery because of the small number of patients included in this subgroup. Therefore, we have launched a prospective, multicenter, large-scale RCT to compare PCS followed by HIPEC with PCS alone for stage III epithelial ovarian cancer.

## Conclusions

In this study, the PCS with HIPEC treatment approach was associated with better long-term survival and was not associated with postoperative severe morbidity and mortality. When complete PCS is possible, this approach can be a valuable therapy among patients with stage III epithelial ovarian cancer.

## References

[1] Hennessy BT, Coleman RL, Markman M Ovarian cancer. Lancet. 2009;374(9698):1371-1382. doi:10.1016/S0140-6736(09)61338-6 19793610

[2] Siegel RL, Miller KD, Jemal A Cancer statistics, 2017. CA Cancer J Clin. 2017;67(1):7-30. doi:10.3322/caac.21387 28055103

[3] Wright AA, Bohlke K, Armstrong DK, Neoadjuvant chemotherapy for newly diagnosed, advanced ovarian cancer: Society of Gynecologic Oncology and American Society of Clinical Oncology clinical practice guideline. J Clin Oncol. 2016;34(28):3460-3473. doi:10.1200/JCO.2016.68.6907 27502591 PMC5512594

[4] Vergote I, Tropé CG, Amant F, ; European Organization for Research and Treatment of Cancer-Gynaecological Cancer Group; NCIC Clinical Trials Group Neoadjuvant chemotherapy or primary surgery in stage IIIC or IV ovarian cancer. N Engl J Med. 2010;363(10):943-953. doi:10.1056/NEJMoa0908806 20818904

[5] Kehoe S, Hook J, Nankivell M, Primary chemotherapy versus primary surgery for newly diagnosed advanced ovarian cancer (CHORUS): an open-label, randomised, controlled, non-inferiority trial. Lancet. 2015;386(9990):249-257. doi:10.1016/S0140-6736(14)62223-6 26002111

[6] van Driel WJ, Koole SN, Sikorska K, Hyperthermic intraperitoneal chemotherapy in ovarian cancer. N Engl J Med. 2018;378(3):230-240. doi:10.1056/NEJMoa1708618 29342393

[7] Armstrong DK, Bundy B, Wenzel L, ; Gynecologic Oncology Group Intraperitoneal cisplatin and paclitaxel in ovarian cancer. N Engl J Med. 2006;354(1):34-43. doi:10.1056/NEJMoa052985 16394300

[8] Chiva LM, Gonzalez-Martin A A critical appraisal of hyperthermic intraperitoneal chemotherapy (HIPEC) in the treatment of advanced and recurrent ovarian cancer. Gynecol Oncol. 2015;136(1):130-135. doi:10.1016/j.ygyno.2014.11.072 25434634

[9] van de Vaart PJ, van der Vange N, Zoetmulder FA, Intraperitoneal cisplatin with regional hyperthermia in advanced ovarian cancer: pharmacokinetics and cisplatin-DNA adduct formation in patients and ovarian cancer cell lines. Eur J Cancer. 1998;34(1):148-154. doi:10.1016/S0959-8049(97)00370-5 9624250

[10] Bakrin N, Bereder JM, Decullier E, ; FROGHI (FRench Oncologic and Gynecologic HIPEC) Group Peritoneal carcinomatosis treated with cytoreductive surgery and Hyperthermic Intraperitoneal Chemotherapy (HIPEC) for advanced ovarian carcinoma: a French multicentre retrospective cohort study of 566 patients. Eur J Surg Oncol. 2013;39(12):1435-1443. doi:10.1016/j.ejso.2013.09.030 24209430

[11] Cascales-Campos PA, Gil J, Gil E, Treatment of microscopic disease with hyperthermic intraoperative intraperitoneal chemotherapy after complete cytoreduction improves disease-free survival in patients with stage IIIC/IV ovarian cancer. Ann Surg Oncol. 2014;21(7):2383-2389. doi:10.1245/s10434-014-3599-4 24599409

[12] Cascales-Campos P, López-López V, Gil J, Hyperthermic intraperitoneal chemotherapy with paclitaxel or cisplatin in patients with stage III-C/IV ovarian cancer: is there any difference? Surg Oncol. 2016;25(3):164-170. doi:10.1016/j.suronc.2016.05.010 27566018

[13] Di Giorgio A, De Iaco P, De Simone M, Cytoreduction (peritonectomy procedures) combined with hyperthermic intraperitoneal chemotherapy (HIPEC) in advanced ovarian cancer: retrospective Italian multicenter observational study of 511 cases. Ann Surg Oncol. 2017;24(4):914-922. doi:10.1245/s10434-016-5686-1 27896512 PMC5339330

[14] Bristow RE, Chi DS Platinum-based neoadjuvant chemotherapy and interval surgical cytoreduction for advanced ovarian cancer: a meta-analysis. Gynecol Oncol. 2006;103(3):1070-1076. doi:10.1016/j.ygyno.2006.06.025 16875720

[15] Japanese Research Society for Gastric Cancer *Japanese Classification of Gastric Carcinoma* 1st English ed Kanehara and Co. Ltd; 1995.

[16] Ozols RF, Bundy BN, Greer BE, ; Gynecologic Oncology Group Phase III trial of carboplatin and paclitaxel compared with cisplatin and paclitaxel in patients with optimally resected stage III ovarian cancer: a Gynecologic Oncology Group study. J Clin Oncol. 2003;21(17):3194-3200. doi:10.1200/JCO.2003.02.153 12860964

[17] Markman M, Bundy BN, Alberts DS, Phase III trial of standard-dose intravenous cisplatin plus paclitaxel versus moderately high-dose carboplatin followed by intravenous paclitaxel and intraperitoneal cisplatin in small-volume stage III ovarian carcinoma: an intergroup study of the Gynecologic Oncology Group, Southwestern Oncology Group, and Eastern Cooperative Oncology Group. J Clin Oncol. 2001;19(4):1001-1007. doi:10.1200/JCO.2001.19.4.1001 11181662

[18] Alberts DS, Liu PY, Hannigan EV, Intraperitoneal cisplatin plus intravenous cyclophosphamide versus intravenous cisplatin plus intravenous cyclophosphamide for stage III ovarian cancer. N Engl J Med. 1996;335(26):1950-1955. doi:10.1056/NEJM199612263352603 8960474

[19] Bristow RE, Tomacruz RS, Armstrong DK, Trimble EL, Montz FJ Survival effect of maximal cytoreductive surgery for advanced ovarian carcinoma during the platinum era: a meta-analysis. J Clin Oncol. 2002;20(5):1248-1259. doi:10.1200/JCO.2002.20.5.1248 11870167

[20] du Bois A, Reuss A, Pujade-Lauraine E, Harter P, Ray-Coquard I, Pfisterer J Role of surgical outcome as prognostic factor in advanced epithelial ovarian cancer: a combined exploratory analysis of 3 prospectively randomized phase 3 multicenter trials: by the Arbeitsgemeinschaft Gynaekologische Onkologie Studiengruppe Ovarialkarzinom (AGO-OVAR) and the Groupe d’Investigateurs Nationaux Pour les Etudes des Cancers de l’Ovaire (GINECO). Cancer. 2009;115(6):1234-1244. doi:10.1002/cncr.24149 19189349

[21] Orr B, Edwards RP Diagnosis and treatment of ovarian cancer. Hematol Oncol Clin North Am. 2018;32(6):943-964. doi:10.1016/j.hoc.2018.07.010 30390767

[22] Chua TC, Robertson G, Liauw W, Farrell R, Yan TD, Morris DL Intraoperative hyperthermic intraperitoneal chemotherapy after cytoreductive surgery in ovarian cancer peritoneal carcinomatosis: systematic review of current results. J Cancer Res Clin Oncol. 2009;135(12):1637-1645. doi:10.1007/s00432-009-0667-4 19701772 PMC11844777

[23] Kireeva GS, Gafton GI, Guseynov KD, HIPEC in patients with primary advanced ovarian cancer: Is there a role? a systematic review of short- and long-term outcomes. Surg Oncol. 2018;27(2):251-258. doi:10.1016/j.suronc.2018.05.006 29937179

[24] Cowan RA, O’Cearbhaill RE, Zivanovic O, Chi DS Current status and future prospects of hyperthermic intraoperative intraperitoneal chemotherapy (HIPEC) clinical trials in ovarian cancer. Int J Hyperthermia. 2017;33(5):548-553. doi:10.1080/02656736.2017.1283066 28092994 PMC5776684

[25] Cavaliere D, Cirocchi R, Coccolini F, ; Italian Society of Surgical Oncology (SICO); Italian Society of Obstetrics and Gynaecology (SIGO); Italian Association of Hospital Obstetricians and Gynaecologists (AOGOI); Italian Association of Medical Oncology (AIOM) 1st Evidence-based Italian consensus conference on cytoreductive surgery and hyperthermic intraperitoneal chemotherapy for peritoneal carcinosis from ovarian cancer. Tumori. 2017;103(6):525-536. doi:10.5301/tj.5000623 28430350

[26] Bonnot PE, Piessen G, Kepenekian V, ; FREGAT and BIG-RENAPE Networks Cytoreductive surgery with or without hyperthermic intraperitoneal chemotherapy for gastric cancer with peritoneal metastases (CYTO-CHIP study): a propensity score analysis. J Clin Oncol. 2019;37(23):2028-2040. doi:10.1200/JCO.18.01688 31084544

[27] Elias D, Lefevre JH, Chevalier J, Complete cytoreductive surgery plus intraperitoneal chemohyperthermia with oxaliplatin for peritoneal carcinomatosis of colorectal origin. J Clin Oncol. 2009;27(5):681-685. doi:10.1200/JCO.2008.19.7160 19103728

[28] Verwaal VJ, van Ruth S, de Bree E, Randomized trial of cytoreduction and hyperthermic intraperitoneal chemotherapy versus systemic chemotherapy and palliative surgery in patients with peritoneal carcinomatosis of colorectal cancer. J Clin Oncol. 2003;21(20):3737-3743. doi:10.1200/JCO.2003.04.187 14551293

[29] Chua TC, Moran BJ, Sugarbaker PH, Early- and long-term outcome data of patients with pseudomyxoma peritonei from appendiceal origin treated by a strategy of cytoreductive surgery and hyperthermic intraperitoneal chemotherapy. J Clin Oncol. 2012;30(20):2449-2456. doi:10.1200/JCO.2011.39.7166 22614976

[30] Cascales PA, Gil J, Galindo PJ, Machado F, Frutos IM, Paricio PP Heterogeneity in patients and methods: a problem for hyperthermic intraoperative intraperitoneal chemotherapy (HIPEC) in ovarian carcinoma. Eur J Obstet Gynecol Reprod Biol. 2011;158(2):361-362. doi:10.1016/j.ejogrb.2011.04.036 21676527

[31] Jewell A, McMahon M, Khabele D Heated intraperitoneal chemotherapy in the management of advanced ovarian cancer. Cancers (Basel). 2018;10(9):E296. doi:10.3390/cancers10090296 30200420 PMC6162496

[32] Muller M, Chérel M, Dupré PF, Gouard S, Collet M, Classe JM Cytotoxic effect of hyperthermia and chemotherapy with platinum salt on ovarian cancer cells: results of an in vitro study. Eur Surg Res. 2011;46(3):139-147. doi:10.1159/000324395 21372578

[33] Katz MH, Barone RM The rationale of perioperative intraperitoneal chemotherapy in the treatment of peritoneal surface malignancies. Surg Oncol Clin N Am. 2003;12(3):673-688. doi:10.1016/S1055-3207(03)00034-6 14567024

[34] Spiliotis J, Halkia E, Lianos E, Cytoreductive surgery and HIPEC in recurrent epithelial ovarian cancer: a prospective randomized phase III study. Ann Surg Oncol. 2015;22(5):1570-1575. doi:10.1245/s10434-014-4157-9 25391263

[35] Gerestein CG, Damhuis RA, Burger CW, Kooi GS Postoperative mortality after primary cytoreductive surgery for advanced stage epithelial ovarian cancer: a systematic review. Gynecol Oncol. 2009;114(3):523-527. doi:10.1016/j.ygyno.2009.03.011 19344936

